# Integrating pleural PD-1^+^CD8^+^ T cell as a complement variable into LENT score to assess patients with lung adenocarcinoma complicated with MPE

**DOI:** 10.3389/fimmu.2026.1630590

**Published:** 2026-02-19

**Authors:** Weizhen Jiang, Leilei Lv, Yaoxin Zhang, Qiuxia Qu, Cheng Chen

**Affiliations:** 1 Department of Respiratory and Critical Medicine, The First Affiliated Hospital of Soochow University, Suzhou, China; 2 Clinical Immunology Laboratory, The First Affiliated Hospital of Soochow University, Suzhou, China

**Keywords:** CD8^+^ T cell, LENT score, malignant pleural effusion, PD-1, prognosis

## Abstract

**Introduction:**

Malignant pleural effusion (MPE) is a common complication of advanced non-small cell lung cancer (NSCLC), particularly in lung adenocarcinoma, and is associated with poor prognosis. A better understanding of the role of PD-1^+^CD8^+^ T cells in the pleural environment and their relevance to patient survival could facilitate better clinical decision-making.

**Methods:**

We performed a cohort study involving NSCLC patients with MPE. The abundance of pleural PD-1^+^CD8^+^ T cells was measured using flow cytometry. We also assessed the presence of epidermal growth factor receptor (EGFR) mutations and programmed death-ligand 1 (PD-L1) expression in the pleural fluid. The LENT score, a known prognostic tool, was combined with pleural PD-1^+^CD8^+^ T cell abundance to develop a novel scoring system, the Immuno-LENT score. The model’s performance was validated using the bootstrap method and concordance index (C-index) calculation.

**Results:**

We found that PD-1^+^CD8^+^ T cells were present in the pleural fluid of all patients with MPE. Notably, the abundance of these cells was influenced by EGFR mutations, while PD-L1 expression had little effect. Patients with a higher abundance of pleural PD-1^+^CD8^+^ T cells also exhibited higher LENT scores, correlating with poorer survival. The Immuno-LENT score, incorporating both the LENT score and pleural PD-1^+^CD8^+^ T cell abundance, was found to be an independent prognostic factor. The model showed strong statistical robustness with a high C-index.

**Discussion:**

The combination of pleural PD-1^+^CD8^+^ T cells with the LENT score offers a more accurate prognostic tool for survival prediction in NSCLC patients with MPE. Our findings suggest that the Immuno-LENT score could guide clinical management and inform therapeutic decisions for these patients, improving patient outcomes by tailoring interventions based on a more comprehensive biomarker profile.

## 1 Introduction

Malignant pleural effusion (MPE) is a refractory complication mostly secondary to advanced lung cancer, usually accompanied by a dismal survival (1, 2). Despite the recent advancement in therapeutic strategies, including target therapy and immunotherapy, a non-negligible portion of patients lost the benefits they gained due to non-sensitive oncogenic epidermal growth factor receptor (EGFR) mutations, or non-responsiveness to immune checkpoint inhibitors (3). As such, the development of new biomarkers and tools to predict the prognosis of lung cancer-associated patients with MPE has become a pressing need.

Cellular immunity plays a major role in tumor microenvironment (TME) and deserves attention (4). CD8^+^ T cells mainly residing in the TME play crucial roles in terms of immune surveillance and antitumor immune response. Though CD8^+^ tumor-infiltrating lymphocytes (TILs) have a decisive influence on antitumor immunity, these cells remained highly heterogeneous such that many tumor-specific CD8^+^ TILs expressing high levels of PD-1 were probably proven to be dysfunctional and exhausted, owing to metabolic and functional alterations, reduced secretion of effector cytokines, decreased cell viability and proliferation, and increased rate of apoptosis, which ultimately renders deficient antitumor activity (5). MPE is a complex microenvironment containing a plethora of immune and tumor signals (6). Similarly, the TME of MPE is generally immunosuppressive with decreased frequencies of CD8^+^ T cells and altered subgroups (7).

Clinically, accurate prognostic stratification is crucial for patients with MPE, as it informs treatment prioritization and clinical decision-making. The LENT score, proposed by Clive et al. in 2014, is the first validated prognostic scoring system specifically developed for MPE (8). This score integrates four readily available clinical parameters: pleural fluid lactate dehydrogenase (LDH), Eastern Cooperative Oncology Group Performance Status (ECOG PS), blood neutrophil-to-lymphocyte ratio (NLR), and tumor type. Based on the total score, patients are stratified into low-, moderate-, and high-risk groups with distinct survival expectations (8).

Despite its widespread clinical use, the LENT score was established before the era of modern targeted therapy and immunotherapy and does not account for tumor molecular characteristics or immune features within the pleural microenvironment. As systemic treatment strategies for advanced lung cancer have rapidly evolved, the predictive accuracy of traditional clinical scoring systems may be challenged, highlighting the need for refined prognostic models that incorporate biologically relevant markers.

In the present study, we evaluated the abundance of PD-1+CD8+ T cells in MPE and investigated its association with diverse clinicopathological characteristics. Furthermore, we aimed to refine the traditional LENT score by integrating pleural PD-1^+^CD8^+^ T cells to establish a novel prognostic model—the Immuno-LENT score—for patients with non-small cell lung cancer (NSCLC) with MPE. To ensure the statistical rigor and reliability of this novel scoring system, we also performed comprehensive internal validation to assess its predictive performance and stability. Ultimately, this study seeks to provide a robust, valuable, and applicable tool to guide optimal clinical management and predict overall survival (OS).

## 2 Materials and methods

### 2.1 Patients

An observational analysis of consecutive patients with lung cancer who underwent thoracentesis for MPE was conducted at the First Affiliated Hospital of Soochow University between April 2019 and July 2021. The inclusion criteria were as follows (1): at least 18 years of age (2); pathologically confirmed lung cancer (3); presentation of histocytologically or cytologically confirmed MPE (4); received systemic treatment following the initial MPE event, which was decided by the multidisciplinary cancer board of our hospital according to the guidelines; and (5) all clinical information and follow-up data accessible. Patients with other tumors and those with incomplete data were excluded. This study was approved by the Ethics Committee of the First Affiliated Hospital of Soochow University (No. 2022-1085) and was conducted in accordance with the Declaration of Helsinki. All participants provided informed consent, and informed consent was obtained prior to the collection of samples.

### 2.2 Data collection and LENT score

Data on each patient’s clinical traits, laboratory results, and treatment details were obtained from the electronic inpatient record system. Clinical characteristics included sex, age, pathology, ECOG PS, tumor proportion score (TPS) of programmed death-ligand 1 (PD-L1), and oncogenic driver mutations. Baseline peripheral blood (PB) indicators included neutrophils, lymphocytes, and the NLR. The location of pleural fluid and pleural fluid LDH levels were also recorded.

The LENT score for each patient was calculated according to the original scoring system proposed by Clive et al. The score consists of four components: LDH level, ECOG PS, NLR, and tumor type. Importantly, lung cancer is assigned two points within the tumor-type category.

Follow-up data were available for all patients for at least 12 months. Survival outcomes were compared between the two LENT risk categories.

### 2.3 Isolation of lymphocytes from PB and MPE

Fresh MPE samples from 76 patients and paired PB from 12 patients were also collected. Peripheral blood mononuclear cells (PBMCs) were separated from venous blood using Ficoll-Paque™ PLUS (GE Healthcare, Sweden) by gradient centrifugation at 1,500 rpm for 5 min. Lymphocytes from malignant pleural fluid were sent to the laboratory within 1 h. Erythrocytes were removed from the pleural effusion by erythrocyte lysis (BioLegend, USA), and the remaining pleural fluid was centrifuged at 1,800 rpm for 5 min. After washing twice with phosphate-buffered saline (PBS), the separated cells were resuspended in 1 mL of RPMI 1640 medium (Gibco, USA).

### 2.4 Analysis of PD-1 on CD8^+^ T cells

For phenotype assessment, cells were suspended in 1.0 mL of RPMI 1640 per 3×10^6^ cells prior to immunostaining. Cells were then incubated with fluorochrome-conjugated antibodies and FVD506 (Fixable Viability Dye eFlour™ 506, eBioscience). In detail, cells were stained with FVD506, CD8a (FITC anti-human CD8a, eBioscience), PD-1 (APC anti-human PD-1, eBioscience), and CD45 (PE-cy7 anti-human CD45, eBioscience) antibodies and incubated on ice for 25 min in the dark. Subsequently, the cells were characterized by flow cytometry analysis (BD LSRFortessa, USA; Thermo-life Attune NxT, USA). Lymphocyte subpopulations were identified by position on forward-scatter (FSC) and side-scatter (SSC) plots. Viable cells were determined by Fixable Viability Dye eFlour™ 506 negative gating. Then, CD8^+^ T cells were gated from the CD45^+^ subsets. During analysis, the percentage of positive cells was recorded.

### 2.5 Determination of the cutoff value for PD-1^+^CD8^+^ T cells

To determine the optimal cutoff value for the proportion of PD-1^+^CD8^+^ T cells in pleural effusion, we used the X-tile software (Yale University), which systematically evaluates all possible divisions of a continuous biomarker using log-rank testing in survival analysis. The software identifies the threshold that produces the greatest separation between survival curves. Based on the algorithmically determined optimal cutoff point, patients were stratified into low and high pleural PD-1^+^CD8^+^ T cell groups for subsequent survival comparison.

### 2.6 Multivariate Cox analysis for prognostic factors

The impact of LENT score, clinical characteristics, and pleural PD-1^+^CD8^+^ T cell on the OS of patients with lung adenocarcinoma complicated with MPE was assessed using univariate and multivariate Cox proportional hazards models. Variables showing statistical significance or borderline significance in the univariate analysis were entered into the multivariate model.

### 2.7 Development of the Immuno-LENT scoring system

The prognostic nomogram (Immuno-LENT score) was constructed using the R programming language and environment (http://www.r-project.org/). It was developed based on the significant predictors identified in the multivariable Cox regression. On the points scale, a specific number of risk points was assigned to each prognostic parameter according to its regression coefficient. A total score was obtained by summing the points for each parameter. Based on the total scores, patients were stratified into low-, moderate-, and high-risk groups for survival comparison.

### 2.8 Internal validation of the scoring system

To comprehensively evaluate the discriminative ability and robustness of the Immuno-LENT score, we first calculated Harrell’s concordance index (C-index), where a value exceeding 0.7 generally indicates good model consistency. Given the limited sample size, we further performed internal validation using the bootstrap resampling technique (1,000 resamples) to derive a bias-corrected C-index; a minimal difference between the original and bias-corrected values serves as a key indicator of model stability and minimal overfitting. Additionally, time-dependent receiver operating characteristic (ROC) curves were generated to assess predictive accuracy at specific time points (3, 6, and 12 months), with an area under the curve (AUC) greater than 0.7 typically considered to indicate high predictive accuracy.

### 2.9 Statistical analysis

Comparisons of continuous variables between different cohorts were performed using the Mann–Whitney *U*-test. Kaplan–Meier curves and log-rank tests were employed to evaluate survival differences. Variables with a *p*-value less than 0.1 in the univariate analysis were included in the multivariable Cox analysis, and the results are presented as hazard ratios (HRs) and 95% confidence intervals (CIs). Statistical significance was defined as a two-tailed *p*-value < 0.05.

## 3 Results

### 3.1 Baseline characteristics of patients

A summary of patient characteristics is presented in **Table 1**. A total of 76 eligible patients were recruited, including 46 male and 30 female patients, with a mean age of 66.8 years (range: 36–92 years). Among these patients, 89.5% were diagnosed with lung adenocarcinoma, and 10.5% were diagnosed with non-adenocarcinoma. Nearly all patients with lung adenocarcinoma (64/68) underwent EGFR testing and 35 harbored EGFR mutations. Notably, 64.5% (49/76) of the patients were diagnosed with MPE during the first episode of lung cancer, while the remaining 27 patients displayed MPE as a progressive or recurrent form of the disease.

**Table 1:** Demographic characteristics of patients with non-small cell lung cancer with MPE.

Variable	Number (%)
Gender	
Male	46 (60.5)
Female	30 (39.5)
Age (years)	
<65	36 (47.4)
>65	40 (52.6)
Pathology	
Adenocarcinoma cancer	68 (89.5)
EGFR^mu^	35
EGFR^wt^	29
Unknown	4
Non-adenocarcinoma cancer	8 (10.5)
Squamous cancer	6
Adenosquamous cancer	2
Recurrent MPE	
Yes	27 (38.2)
No	49 (61.8)

EGFR^mu^, EGFR mutations; EGFR^wt^, EGFR wild type.

### 3.2 Accumulation of PD-1^+^CD8^+^ T cells in MPE

**Figure 1:**
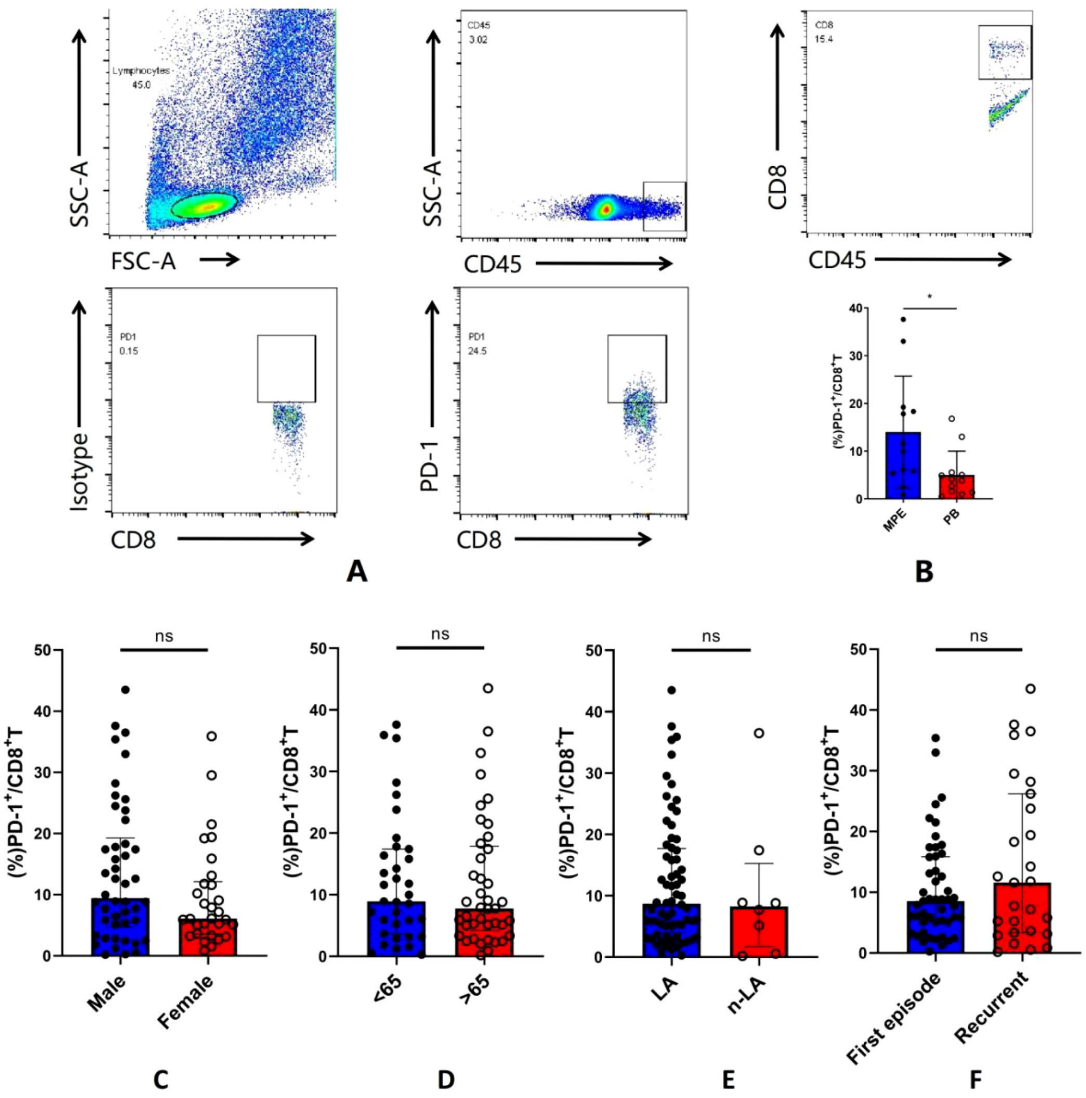
Accumulation of PD-1^+^CD8^+^ T cells in MPE. **(A)** Representative image of flow cytometry analysis about PD-1^+^CD8^+^ T cells in MPE. **(B)** The frequency of PD-1^+^CD8^+^ T cells was higher in MPE compared with matched PB. **(C–F)** Determination of pleural PD-1^+^CD8^+^ T cells in patients with different clinical settings according to gender, age, histologic cell types, and disease status. *P<0.05.

As CD8^+^ T cells with high levels of PD-1 have been reported to be abundant in TMEs, we extended the data by examining the frequency of PD-1^+^CD8^+^ T cells in MPE and their matched PB. First, the data showed that the frequency of PD-1^+^CD8^+^ T cells was higher in MPE than in matched PB (median: 14.5% *vs*. 3.99%, *p* < 0.05, **Figures 1A, B**). This finding suggests that induction and education within MPE would drive the expression of PD-1 on CD8^+^ T cells. We then determined the number of pleural PD-1^+^CD8^+^ T cells in patients with NSCLC-MPE with different clinical settings. As shown in **Figures 1C–F**, no significant difference in pleural PD-1^+^CD8^+^ T cells was observed between the male group compared with the female group (median: 9.44% *vs*. 6.09%, *p* > 0.05). The proportion of pleural PD-1^+^CD8^+^ T cells in the elderly group was similar to that in the younger group (median: 7.75% *vs*. 8.90%, *p* > 0.05). In addition, lung adenocarcinoma-related MPE displayed levels of pleural PD-1^+^CD8^+^ T cells that were comparable to those in non-lung adenocarcinoma (median: 8.69% *vs*. 8.25%, *p* > 0.05). Regarding disease status, a relatively but insignificantly higher abundance of pleural PD-1^+^CD8^+^ T cells was observed in recurrent NSCLC than in the first MPE (median: 11.6% *vs*. 8.55%, *p* > 0.05). Together, these findings indicate that PD-1^+^CD8^+^ T cells are ubiquitously present in the MPE of patients with NSCLC.

### 3.3 Pleural PD-1^+^CD8^+^ T cells correlate with EGFR mutation status but not with PD-L1 expression

**Figure 2:**
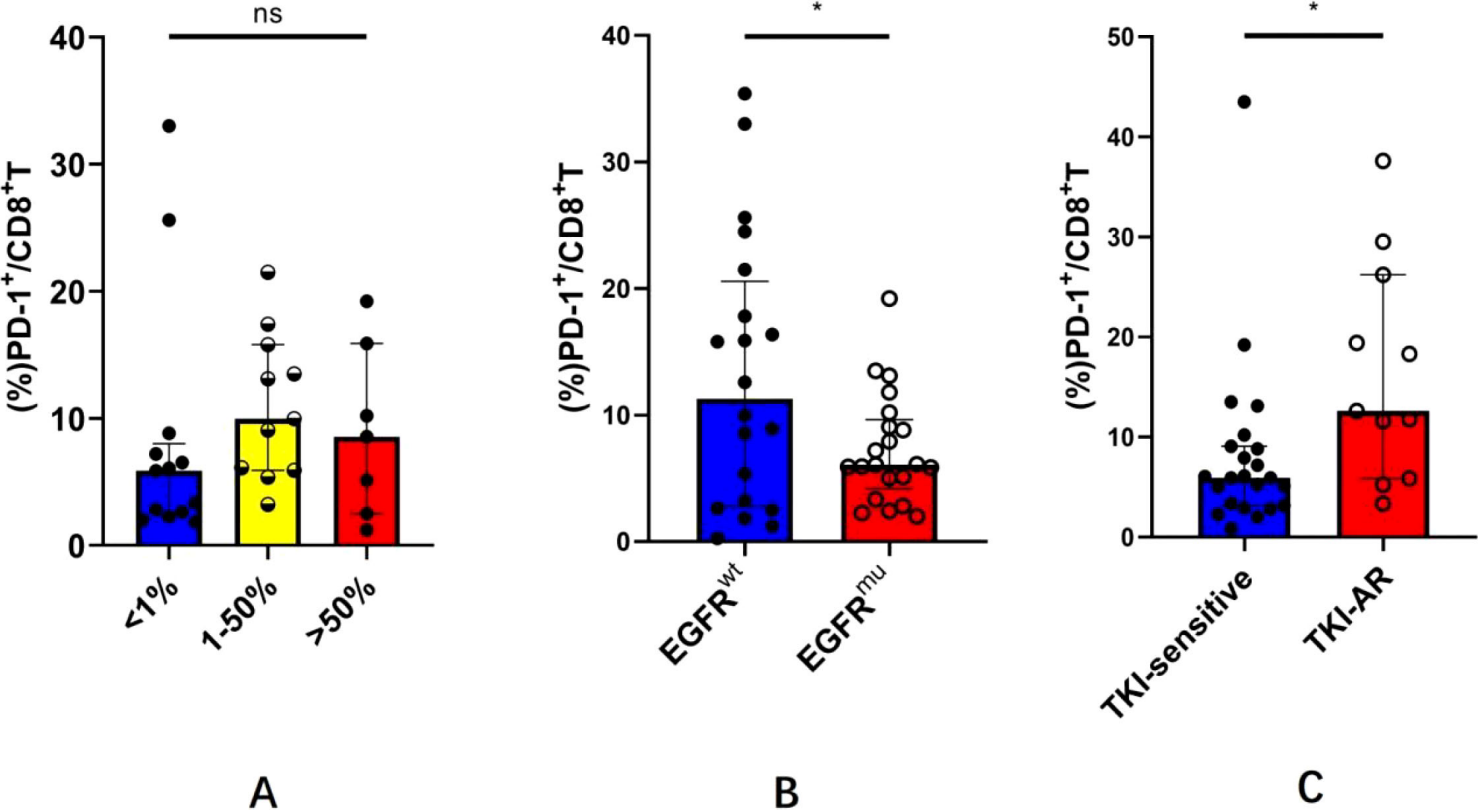
Pleural PD-1^+^CD8^+^ T cells in EGFR mutation or tumoral-PD-L1 expression defined MPE. **(A)** One-way ANOVA test was used to compare the abundance of pleural PD-1^+^CD8 ^+^T cells among the subgroup with PD-L1 TPS <1%, 1%–50%, and >50%. **(B)** Mann–Whitney *U*-test was used to analyze the PD-1^+^CD8^+^ T cells in MPE from EGFR^mu^-MPE and EGFR^wt^-MPE. **(C)** Mann–Whitney *U*-test was used to analyze the PD-1^+^CD8^+^ T cells in MPE with TKI-acquired resistance and TKI-sensitive MPE EGFR^mu^ from subgroups. *P<0.05.

Next, we investigated the abundance of pleural PD-1^+^CD8^+^ T cells in the context of tumoral-PD-L1 expression or EGFR mutations. Based on the TPS of tumoral PD-L1, it was found that there was no significant difference in the frequency of pleural PD-1^+^CD8^+^ T cells among the subgroup with TPS < 1%, 1%–50%, and >50% (median: 5.85% *vs*. 9.98% *vs*. 8.55%, *p* > 0.05, **Figure 2A**). As shown in **Figure 2B**, the frequency of pleural PD-1^+^CD8^+^ T cells in EGFR^mu^-MPE was significantly lower than that in EGFR^wt^-MPE (7.32% ± 4.36% *vs*. 13.15% ± 10.66%, *p* < 0.05). Within the EGFR^mu^-MPE subgroup, the frequency of pleural PD-1^+^CD8^+^ T cells in MPE with TKI-acquired resistance was significantly higher than that in TKI-sensitive MPE (median: 12.60% *vs*. 5.92%, *p* < 0.05, **Figure 2C**). These results demonstrate that pleural PD-1^+^CD8^+^ T cells are more relevant to EGFR gene status, independent of tumor PD-L1 expression.

### 3.4 Evaluating pleural PD-1^+^CD8^+^ T cells as a potential prognostic factor

**Figure 3:**
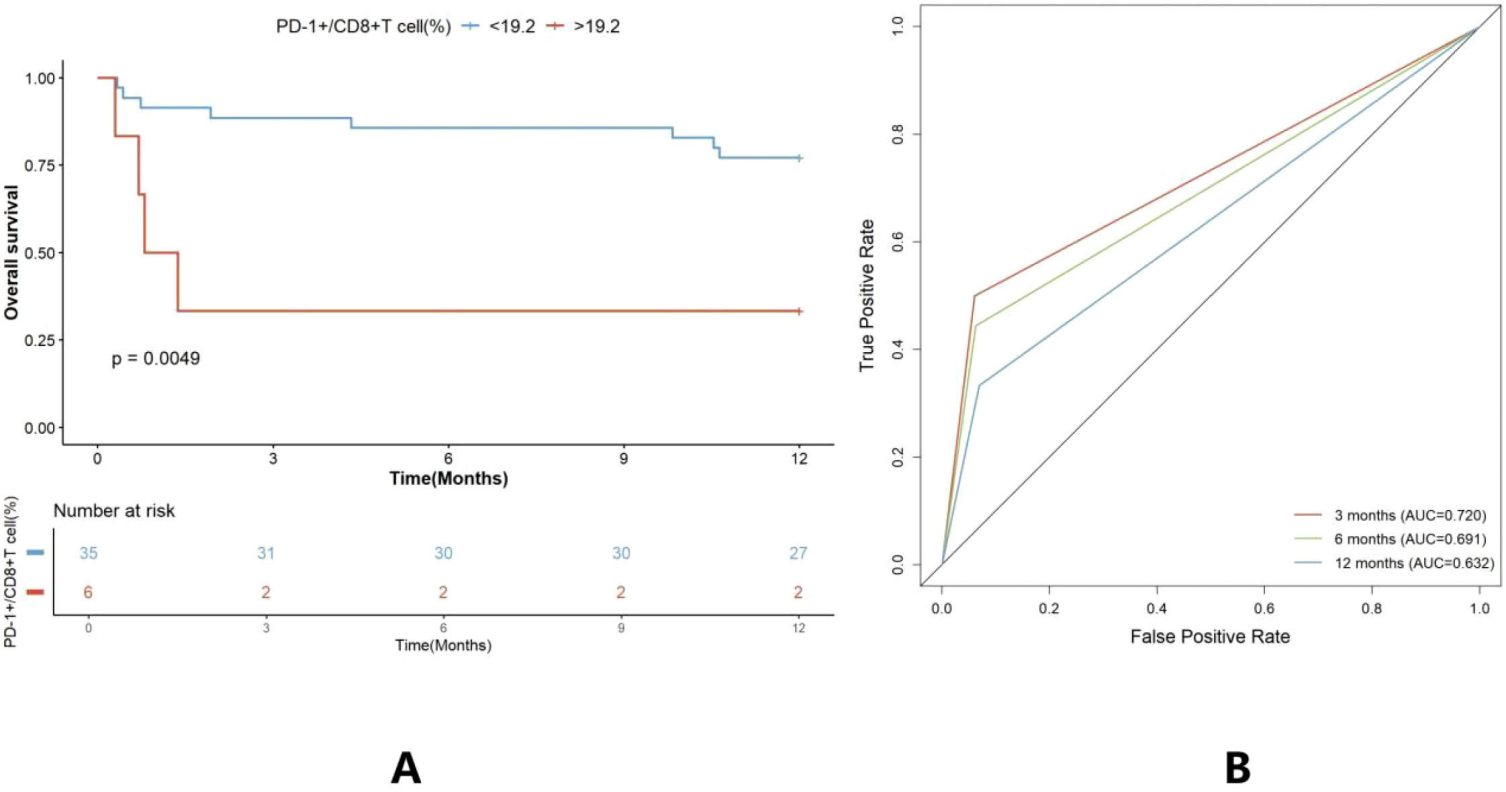
Pleural PD-1^+^CD8^+^ T cells predict prognosis in patients with NSCLC. **(A)** The OS of patients with MPE with high and low pleural PD-1^+^CD8^+^ T cell was analyzed by Kaplan–Meier survival curve. **(B)** The AUCs were 0.720, 0.691, and 0.632 for the pleural PD-1^+^CD8^+^ T cell indicating survival at 3, 6, and 12 months.

To elucidate the clinical implications of pleural PD-1^+^CD8^+^ T cells, we first determined the optimal cutoff value for survival stratification using the X-tile software. The analysis identified 19.2% as the optimal threshold. Based on this cutoff, patients were categorized into low-expression (<19.2%) and high-expression (>19.2%) groups. As shown in **Figure 3A**, patients with higher pleural PD-1^+^CD8^+^ T levels had a significantly worse prognosis than those with lower pleural PD-1^+^CD8^+^ T levels. In detail, patients with lower pleural PD-1^+^CD8^+^ T cell had 88.57%, 85.71%, and 77.14% of the patients survived at 3, 6, and 12 months, respectively. This was better than those with higher pleural PD-1^+^CD8^+^ T cell levels, with 33.33%, 33.33%, and 33.33% survival at 3, 6, and 12 months, respectively. Correspondingly, the AUCs for pleural PD-1^+^CD8^+^ T cells were 0.720, 0.691, and 0.632, indicating survival at 3, 6, and 12 months, respectively (**Figure 3B**).

### 3.5 Integration of pleural PD-1^+^CD8^+^ T cells into LENT score

**Figure 4:**
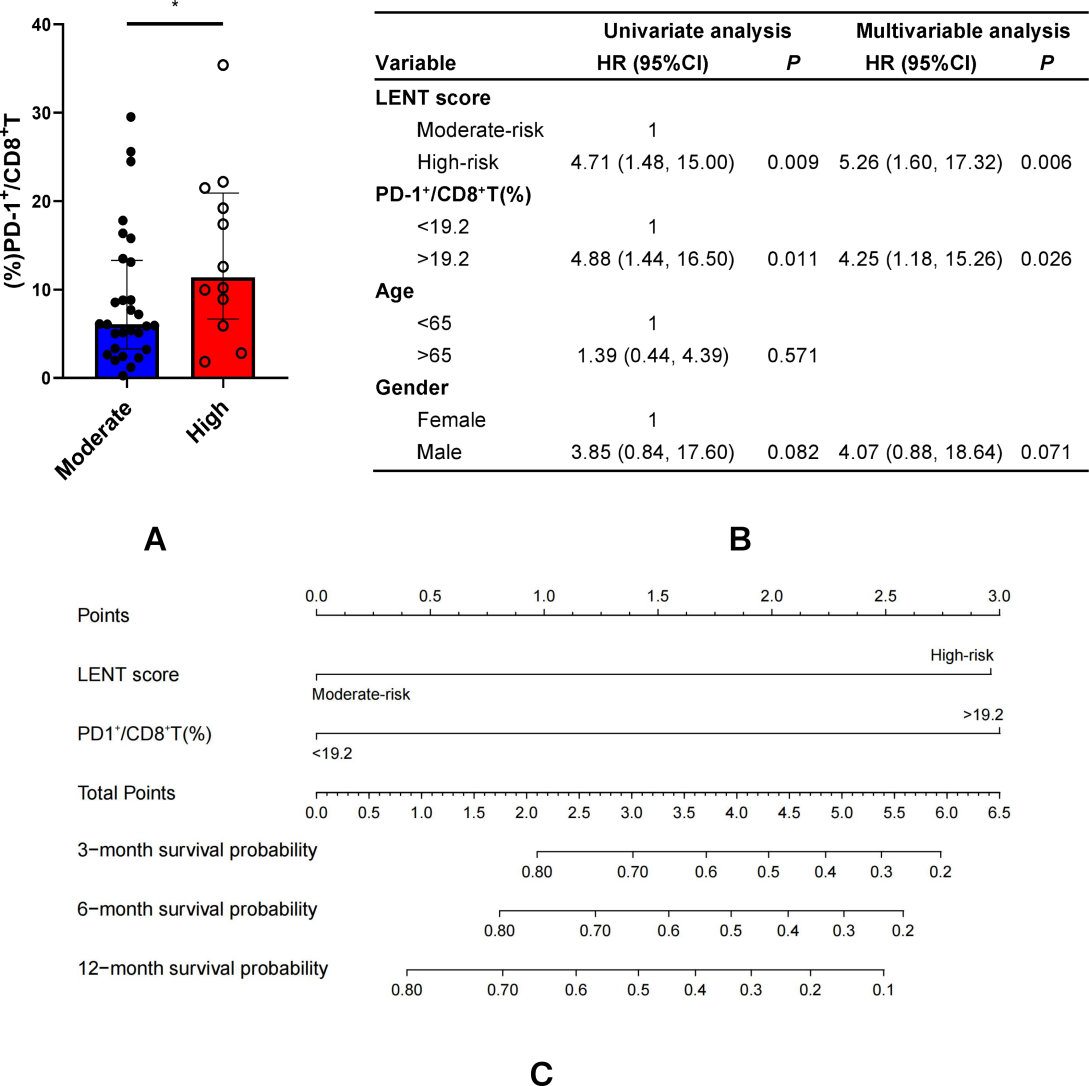
Integration of pleural PD-1^+^CD8^+^ T cells into LENT score. **(A)** Pleural PD-1^+^CD8^+^ T cells were compared between those with a high-risk and those with a moderate-risk LENT score. **(B)** Univariate and multivariate analysis was used to establish the independent variables associated with survival. **(C)** The nomogram was plotted and validated using the R programming language and environment (http://www.r-project.org/). LENT score and pleural PD-1^+^CD8^+^ T cells were selected to establish a novel score (called Immuno-LENT score) to predict the likelihood of survival at 3, 6, and 12 months using the nomogram. *P<0.05.

The LENT scoring system is the first validated prognostic model for MPE. Moreover, patients with lung adenocarcinoma-associated MPE inherently have a minimum total score of 2, meaning that none of the patients in this study could fall into the low-risk LENT category (0–1 points). As a result, patients were categorized into moderate-risk (2–4 points) and high-risk (5–7 points) groups based on their total LENT scores. Those with a high-risk LENT score had an increased frequency of pleural PD-1^+^CD8^+^ T cells compared to those with a moderate-risk LENT score (median: 11.40% *vs*. 6.06%, *p* < 0.05, **Figure 4A**). Based on univariate and multivariate analyses, the LENT score and pleural PD-1^+^CD8^+^ T cells were identified as the variables to maintain independent associations with survival at a predefined cutoff (*p* < 0.1; **Figure 4B**). The LENT score and pleural PD-1^+^CD8^+^ T cells were then selected to establish a novel score (called the Immuno-LENT score) to predict the likelihood of survival at 3, 6, and 12 months using a nomogram. **Figure 4C** shows the scoring system; a proportional number of risk points was assigned to each prognostic parameter, resulting in a score range of 0–2. For ease of interpretation, the patients were divided into low-risk (score 0), moderate-risk (score 1), and high-risk (score 2) prognostic groups (**Table 2**).

**Table 2:** The Immuno-LENT score for patients with NSCLC diagnosed with MPE during the first episode.

Variable	Point
LENT score	
Moderate risk	0
High risk	1
PD-1^+^/CD8^+^ T(%)	
<19.2	0
>19.2	1
Risk categories	Total score
Low risk	0
Moderate risk	1
High risk	2

### 3.6 Comparison of the Immuno-LENT score with the LENT score

**Figure 5:**
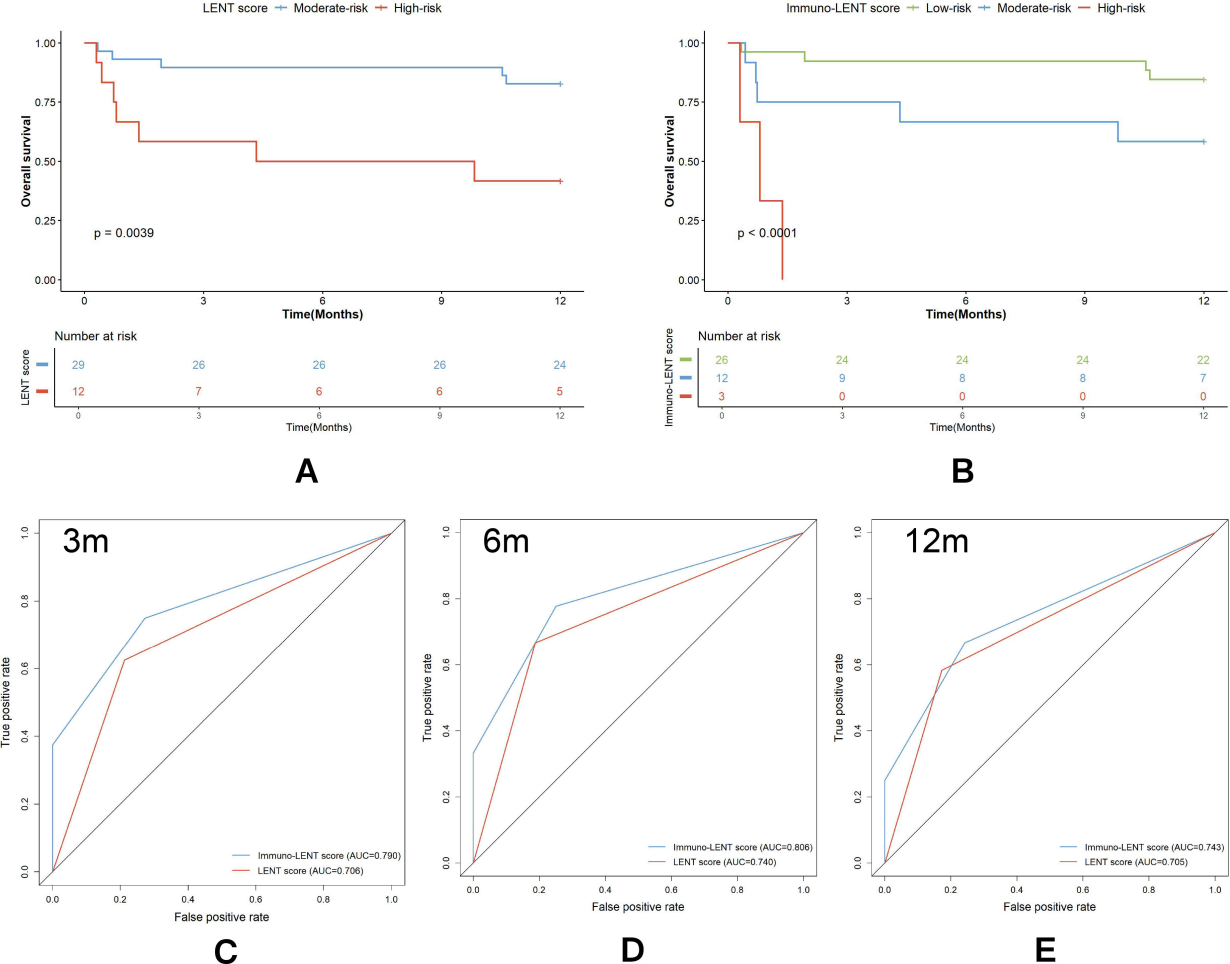
Performance of LENT score and Immuno-LENT score in LA patients with MPE. **(A)** Kaplan–Meier survival curves from patients with MPE with moderate and high LENT score. **(B)** Kaplan–Meier survival curves from patients with MPE with low, moderate, and high Immuno-LENT scores. **(C–E)** Comparison between survival data reported in the original LENT and Immuno-LENT scoring system.

First, newly diagnosed patients with LA-MPE were divided into groups with moderate or high risk using the LENT score, and 89.66%, 89.66%, and 82.76% of the patients with a moderate-risk LENT score survived for 3, 6, and 12 months, respectively (**Figure 5A**). Otherwise, 58.33% of those with a high-risk LENT score survived for 3 months, 50.00% for 6 months, and 41.67% for 12 months. We then explored the potential of the Immuno-LENT score to predict OS in these patients (**Figure 5B**). Of all the patients, 26 patients were assigned into the low-risk group, with 92.31%, 92.31%, and 84.62% surviving at 3, 6, and 12 months, respectively. The survival at 3, 6, and 12 months of the patients in the moderate-risk group was 75.00%, 66.67%, and 58.33%, respectively. The OS of all the patients in the high-risk group was less than 3 months. Correspondingly, the AUCs were 0.790, 0.806, and 0.743 for the Immuno-LENT scores at 3, 6, and 12 months, respectively. These values were consistently higher than those for the original LENT score (0.706, 0.740, and 0.705, respectively) (**Figures 5C–E**), indicating superior predictive efficacy.

### 3.7 Internal validation of the immuno-LENT score

**Figure 6:**
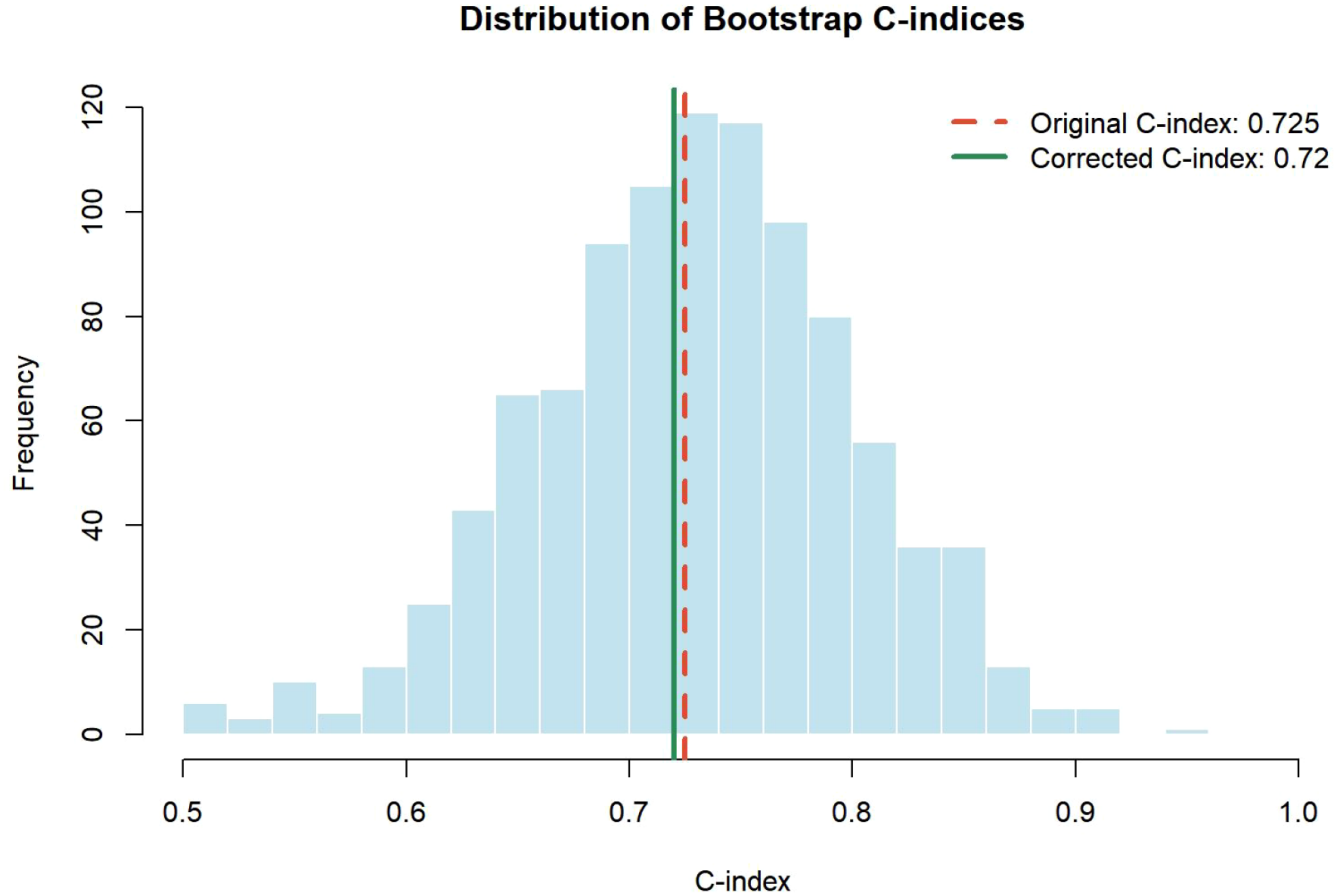
Internal validation of the Immuno-LENT score. The histogram displays the frequency distribution of C-indices derived from 1,000 bootstrap resamples. The red dashed line represents the apparent C-index (0.725), and the green solid line represents the bias-corrected C-index (0.720).

To assess model robustness, internal validation was performed using the bootstrap resampling technique. The Immuno-LENT model achieved an overall C-index of 0.725, with a bias-corrected C-index of 0.720 (**Figure 6**). The negligible difference between these values indicates stable discriminative ability with minimal overfitting, effectively mitigating concerns regarding the limited sample size. Consequently, the integrated Immuno-LENT model demonstrated superior prognostic accuracy and stability compared to both pleural PD-1^+^CD8^+^ T cells alone and the original LENT score.

## 4 Discussion

In the present study, we reported higher levels of PD-1^+^CD8^+^ T cells in MPE compared to PB, which extends the idea that PD-1^+^CD8^+^ T cells represented the defined cell population in MPE. Although it was observed that PD-1^+^CD8^+^ T cells were ubiquitously presented in MPE caused by NSCLC, the abundance of pleural PD-1^+^CD8^+^ T cells was impacted by EGFR mutation. As PD-1 expression is upregulated when T cells are exposed to antigen, the different levels of PD-1 expression may be related to the level of antigen exposure of cancer cells in the pleural effusion (9). Here, we hypothesized that a low TMB presented by EGFR^mu^-MPE results in a lack of immunogenic neo-antigens, which in turn decreases the number of PD-1^+^CD8^+^ T cells in MPE. This result was inconsistent with our previous findings, in which EGFR status impacted the accumulation of CD39^+^CD8^+^ T cells in lung adenocarcinoma-related MPE.

In parallel with these specific cellular findings, the broader integration of pleural biomarkers represents a promising frontier for MPE prognostication. Systematic analyses have identified markers such as VEGF, pH, glucose, and survivin as significant predictors of survival, suggesting that capturing the microenvironmental landscape of the pleural space adds valuable prognostic information (10). Moreover, molecular approaches like cfDNA sequencing are gaining traction as an innovative way to gain insights into tumor biology, identify therapeutic targets, and offer a promising avenue for integrating prognostic models (11). Emerging evidence also indicates that pleural fluid biomarkers may extend beyond survival prediction to guide local management, such as predicting responses to pleurodesis (12). These advances underscore the clinical relevance of exploring specific immune subsets like PD-1^+^CD8^+^ T cells.

Because of its key role in the regulation of T-cell responses, PD-1 was known as an immune checkpoint molecule (13). Previous studies have proven that high PD-1 expression of CD8^+^ T cell in TME is characteristic of depletion (5, 14). Therefore, we hypothesized that the PD-1^+^CD8^+^ T cell in pleural fluid was a potential prognostic factor in patients with NSCLC complicated with MPE, and higher pleural PD-1^+^CD8^+^ T cells were associated with a worse prognosis for patients. However, these findings need to be further confirmed by studies with larger sample sizes.

Clinically, the ability to predict survival helps prioritize therapeutic options for MPE. The LENT scoring system developed in 2014 was the first validated prognostic model in MPE, which predicted patient survival according to pleural fluid LDH, ECOG PS score, type of tumor, and blood NLR (8). However, as oncologic and pleural treatment options develop, immunotherapy and targeted therapy have dramatically improved the prognosis of advanced lung cancer (15, 16). This evolution in the treatment landscape has challenged the precision of historical models. A recent large contemporary retrospective cohort study (2015–2023) revealed that despite significant progress in treatment options, OS after MPE onset has not significantly improved, highlighting a persistent need for refined predictive tools (17). Furthermore, an external validation of established systems demonstrated that the LENT and PROMISE scores showed only moderate discrimination in modern cohorts, underscoring their limitations in the current clinical context (18). Therefore, the original LENT score, which does not account for patients’ modern therapy patterns, might be less accurate in predicting the prognosis of patients with lung cancer with MPE. Here, we integrated pleural PD-1^+^CD8^+^ T cell as a complement variable into LENT score to assess patients with lung adenocarcinoma complicated with MPE. As supported, the refinement by inclusion of pleural PD-1^+^CD8^+^ T cells into LENT score improved its performance in predicting survival among patients with NSCLC complicated with MPE.

It is worth noting that the prevalence of EGFR mutations in patients with NSCLC complicated with MPE was nearly half, who displayed a lower level of pleural PD-1^+^CD8^+^ T cells and who were intended to receive TKI treatment (19, 20). Meanwhile, those with a high-risk LENT score had an increase in the frequency of pleural PD-1^+^CD8^+^ T cells compared to those with a moderate-risk LENT score. We believed that these were the reasons for Immuno-LENT score being different from the original LENT score.

Taken together, our study is subject to limitations, primarily its single-center design and relatively limited sample size; however, the Immuno-LENT model achieved a C-index of 0.725 (95% CI: 0.454–0.996) with a bias-corrected C-index of 0.720, suggesting stable discriminative ability and minimal overfitting despite these constraints. Although immediate external validation was practically challenging because the detection of pleural PD-1^+^CD8^+^ T cells is not currently a routine clinical test, the primary merit and innovation of this study lie in refining the original LENT score, which was established before the widespread adoption of modern immunotherapies and targeted therapies. Consequently, the proposed Immuno-LENT score provides an updated prognostic assessment relevant to the latest advancements in lung cancer treatment, warranting future multi-center collaborations to prospectively validate this system in larger cohorts.

## Data Availability

Due to patient privacy and confidentiality regulations, the raw data underlying this study cannot be publicly shared. The dataset contains sensitive clinical and immunological information protected under ethical guidelines to ensure participant anonymity and compliance with institutional and legal requirements. However, reasonable requests for data access can be considered on a case-by-case basis subject to approval by the corresponding author and relevant ethics committees. Interested researchers are encouraged to contact the corresponding author directly to discuss potential collaborations or data-sharing agreements. Requests to access the datasets should be directed to Cheng Chen, chencheng@suda.edu.cn.
